# Documented Anemia and Recorded Management Patterns Among Major-Surgery Hospitalizations: A Retrospective Real-World Study

**DOI:** 10.3390/healthcare14213742

**Published:** 2026-11-03

**Authors:** Alin Ionescu, Alina Abu-Awwad, Alexandra Mihăilescu, Dorel Săndesc, Raluca Dumache, Nicolae Albulescu, Noemi Mezsaros, Ahmed Abu-Awwad, Anca-Mihaela Bina, Mihai Alexandru Săndesc

**Affiliations:** 1 Doctoral School, “Victor Babeș” University of Medicine and Pharmacy, 300041 Timisoara, Romania; ionescu.alin@umft.ro; 2 Research Center in Anesthesia and Intensive Care (CCATITM), “Victor Babeș” University of Medicine and Pharmacy, 300041 Timisoara, Romania; sandesc.dorel@umft.ro; 3 Department of Obstetrics and Gynecology, “Victor Babeș” University of Medicine and Pharmacy, 300041 Timisoara, Romania; alina.abuawwad@umft.ro (A.A.-A.); lungu.anca@umft.ro (A.-M.B.); 4 Centre of Genomic Medicine, Genetics Discipline, “Victor Babeș” University of Medicine and Pharmacy, 300041 Timisoara, Romania; 5 Center for Ethics in Human Genetic Identifications, “Victor Babeș” University of Medicine and Pharmacy, 300041 Timisoara, Romania; raluca.dumache@umft.ro; 6 Department of Internal Medicine II, Division of Cardiology, Centre for Molecular Research in Nephrology and Vascular Disease, “Victor Babeș” University of Medicine and Pharmacy, 300041 Timisoara, Romania; nicolae.albulescu@umft.ro; 7 Department of Life Sciences, Faculty of Medicine, Vasile Goldiş Western University of Arad, 310045 Arad, Romania; mezsaros.noemi@uvvg.ro; 8 Department XV-Discipline of Orthopedics and Traumatology, “Victor Babeș” University of Medicine and Pharmacy, 300041 Timisoara, Romania; sandesc.mihai@umft.ro; 9 Research Center University Professor Doctor Teodor Sora, “Victor Babeș” University of Medicine and Pharmacy, 300041 Timisoara, Romania

**Keywords:** perioperative anemia, major surgery, Patient Blood Management, ferric carboxymaltose, intravenous iron, blood transfusion, administrative data, retrospective study, hospital resource utilization

## Abstract

**Highlights:**

**What are the main findings?**

• Documented anemia affected approximately 28% of major-surgery hospitalizations in both 2017 and 2018, with substantial variation across surgical categories.
• Recorded ferric carboxymaltose administration decreased in 2018, whereas recorded blood transfusion increased, indicating a shift in anemia-management patterns between the two years.

**What are the implications of the main findings?**

• Administrative hospital data can identify surgical categories with a high burden of documented anemia and marked variation in recorded management practices.
• The findings support prospective clinical audits and the development of standardized, procedure-sensitive Patient Blood Management pathways, while not establishing treatment appropriateness or effectiveness.

**Abstract:**

Background/Objectives: Perioperative anemia is common among patients undergoing major surgery and represents an important component of Patient Blood Management (PBM). However, real-world patterns of anemia documentation and management may vary across surgical settings. This study aimed to evaluate the prevalence and distribution of documented anemia and the recorded use of ferric carboxymaltose (FCM) and blood transfusion among major-surgery hospitalizations, comparing two consecutive years and different surgical categories. Methods: This retrospective observational study included 10,571 adult hospitalizations involving major surgical procedures at a Romanian tertiary emergency hospital between 1 January 2017 and 31 December 2018. Routinely collected administrative data were used to identify anemia diagnoses, recorded FCM administration, red blood cell (RBC) transfusion, and selected hospital resource utilization measures. Anemia diagnoses were checked against hemoglobin results documented in the medical records, and the anemia status was recorded as present or absent; individual hemoglobin values were not retained in the dataset. FCM administration and transfusion were recorded across the entire surgical population and could not be consistently linked to individual hospitalizations with documented anemia. Comparisons between years were performed using Pearson’s chi-square test, with effect sizes expressed as odds ratios and 95% confidence intervals. An exploratory analysis assessed length of stay, intensive care unit stay, and surgical duration according to recorded management patterns in selected orthopedic categories. Results: The study included 5370 hospitalizations in 2017 and 5201 in 2018. Documented anemia was recorded in 1514 (28.2%) and 1439 (27.7%) hospitalizations, respectively, showing a relatively stable overall prevalence but substantial variation across surgical categories. Recorded FCM administration decreased in 2018, whereas recorded blood transfusion increased. These frequencies refer to all surgical hospitalizations and do not necessarily represent interventions administered to the same hospitalizations in which anemia was documented. The relationship between documented anemia and recorded management events varied across procedure groups. Exploratory orthopedic analyses also indicated differences in hospital resource utilization between management groups, although interpretation was limited by small subgroup sizes and incomplete variability data. Conclusions: Documented anemia affected approximately one-quarter of major-surgery hospitalizations, while recorded management patterns differed by year and surgical category. These findings support procedure-sensitive clinical audits and more structured PBM pathways but do not establish treatment appropriateness or effectiveness.

## 1. Introduction

Perioperative anemia is common among patients undergoing major surgery and has been associated with increased transfusion requirements and adverse postoperative outcomes [1,2]. It may result from iron deficiency, chronic inflammation, ongoing blood loss, renal dysfunction, nutritional deficiencies, or a combination of these mechanisms. Because some of these causes are potentially modifiable, contemporary perioperative care emphasizes not only the recognition of anemia but also the identification and management of its underlying mechanism [3].

Patient Blood Management (PBM) was developed around this principle. Rather than relying primarily on allogeneic blood transfusion once anemia becomes clinically significant, PBM promotes a proactive and individualized approach based on early recognition and treatment of anemia, reduction in avoidable blood loss, and optimization of the patient’s physiological tolerance to anemia [4]. Within this framework, iron deficiency deserves particular attention because it is a common and potentially treatable cause of perioperative anemia. Intravenous iron may be considered when oral iron is ineffective or poorly tolerated, or when the interval before surgery is insufficient for an adequate response to oral therapy [5].

Translating PBM recommendations into routine clinical practice remains challenging because patients undergoing major surgery constitute a clinically heterogeneous population. Some patients are assessed several weeks before an elective intervention, providing sufficient time for anemia investigation and correction, whereas others require urgent surgery with limited opportunity for preoperative optimization [6]. Management may also differ according to procedure type, surgical specialty, institutional resources, clinician preferences, and the availability of locally standardized pathways. Consequently, the documentation and management of anemia may vary substantially between surgical settings, even within the same institution [7].

This variability is particularly relevant when PBM-related practice is assessed using routinely collected hospital data. Administrative records may provide valuable information regarding the frequency of documented anemia, the use of selected therapeutic interventions, and differences between surgical categories or study periods [8]. However, such data do not necessarily capture the complete clinical reasoning underlying each management decision. A documented anemia diagnosis recorded intravenous iron administration, and recorded blood transfusion should therefore be interpreted as separate administrative indicators rather than as consecutive stages of an individual treatment pathway [9,10].

Despite these limitations, real-world institutional data can identify clinically relevant variation that may not be evident from overall prevalence estimates alone [11]. Comparing documented anemia with recorded ferric carboxymaltose administration and blood transfusion across major surgical categories may reveal procedure groups with distinct patterns of recognition and management. Such findings cannot establish treatment appropriateness or effectiveness, but they can generate hypotheses, identify priorities for prospective clinical audit, and support the development of more structured and procedure-sensitive PBM pathways [12].

Although laboratory-based anemia criteria and selected biochemical measurements were reported in the previous publication using the same institutional surgical population [13], the present secondary analysis retained only binary anemia status (present/absent), based on the review of the hemoglobin results in the medical records; individual hemoglobin values were not retained in the aggregated dataset. Evaluating these patterns at the institutional level may provide complementary information regarding variation in anemia documentation and management across surgical specialties and study years. Importantly, this approach may help identify surgical categories with a high burden of documented anemia and differences between anemia documentation and recorded management-event frequencies. Such information may help prioritize procedure-specific clinical audits, identify areas requiring more detailed assessment of Patient Blood Management (PBM) practices, and guide the development of targeted institutional quality-improvement initiatives.

Therefore, this study aimed to describe the prevalence and distribution of documented anemia and the recorded use of ferric carboxymaltose and blood transfusion among major-surgery hospitalizations. We also examined the differences between 2017 and 2018 and variation across major surgical categories. A secondary exploratory objective was to describe hospital resource utilization according to recorded anemia-management patterns in selected orthopedic procedure groups.

## 2. Materials and Methods

### 2.1. Study Design and Setting

This retrospective observational study was based on routinely collected administrative data from adult hospitalizations involving major surgical procedures at a large tertiary emergency hospital in Romania. The analysis covered two consecutive calendar years, from 1 January 2017 to 31 December 2018, and evaluated the distribution of documented anemia, recorded ferric carboxymaltose administration, and recorded blood transfusion across major surgical categories.

The 2017–2018 period was selected because it represented the historical institutional dataset available for the present secondary analysis. These data provide a retrospective description of anemia documentation and recorded management practices during a defined period, rather than an assessment of current institutional PBM implementation. Although contemporary PBM recommendations have evolved since the study period, the dataset remains relevant for examining historical variation across surgical categories and identifying methodological and organizational priorities for future clinical audits.

The source dataset was generated from the hospital’s official reports concerning patients discharged from continuous inpatient care and submitted to the Romanian School of Public Health, Management and Professional Development (SNSPDPMS). These records were cross-referenced at the institutional reporting level with medication-consumption data financed through the hospital budget or national healthcare programs and with blood transfusion records covering the same period. Hospital discharge records provided information on surgical hospitalizations, A-DRG classifications, and documented anemia diagnoses, whereas medication-consumption and transfusion records provided the corresponding management-event counts. The data were summarized by calendar year and surgical A-DRG category. Although these sources allowed category-level comparisons, they did not consistently permit hospitalization-level linkage between documented anemia and individual interventions across the entire surgical population. Consequently, documented anemia, FCM administration, and transfusion were analyzed as separate indicators rather than mutually exclusive patient-level treatment categories.

The available data included the number of hospitalizations involving major surgical procedures, Major Diagnostic Category and Diagnosis-Related Group classifications, documented primary or secondary diagnoses of anemia, recorded ferric carboxymaltose administration, recorded blood transfusion, and selected measures of hospital resource utilization. Analyses were performed using both aggregated hospital-level data and more detailed subgroup data available for selected orthopedic categories.

This study represents a secondary analysis of the institutional surgical population previously investigated by Ionescu et al. [13] but addresses distinct research questions using a different analytical dataset. The previous publication focused primarily on perioperative iron-deficiency anemia management, including laboratory-based anemia criteria, intravenous FCM administration, blood transfusion, and selected clinical and biochemical outcomes. In contrast, the present study uses aggregated institutional reporting data to examine the distribution of documented anemia and recorded management events across Major Diagnostic Categories (MDCs) and adjacent Diagnosis-Related Groups (A-DRGs), as well as year-to-year differences in these indicators. Additional contributions include surgical-category-stratified analyses of recorded FCM administration and transfusion and exploratory resource-utilization comparisons in selected orthopedic subgroups. Unlike the earlier analysis, the dataset available for the present study did not retain individual hemoglobin values or complete patient-level treatment information. Anemia diagnoses recorded in the discharge data were checked against hemoglobin results documented in the medical records. Only the binary anemia status (present/absent) was retained; individual hemoglobin values were not available in the aggregated dataset. The findings were interpreted as administrative indicators rather than individual clinical treatment pathways.

### 2.2. Study Population and Classification of Surgical Procedures

The study population comprised 10,571 adult hospitalizations involving major surgical procedures, including 5370 hospitalizations in 2017 and 5201 in 2018.

Major surgical procedures were identified through a structured clinical assessment of the surgical intervention recorded in the institutional hospital database. Procedures were considered eligible when they involved extensive dissection of deep anatomical structures, major organ or vascular manipulation, substantial anatomical resection or reconstruction, or clinically relevant anticipated perioperative blood loss. Surgical complexity was assessed according to the anatomical extent and technical characteristics of the procedure rather than the postoperative outcomes. Superficial interventions, minor procedures, and diagnostic procedures without substantial anatomical intervention were excluded.

Eligible procedures encompassed major orthopedic interventions, including hip and knee arthroplasty and major fracture fixation; complex spinal surgery; major gastrointestinal resections; vascular reconstruction; major hepatobiliary and pancreatic surgery; extensive urological procedures; and major gynecological interventions.

Eligibility was determined independently of postoperative outcomes, actual intraoperative blood loss, transfusion requirements, or length of hospital stay. The same clinical eligibility criteria and selection procedures were applied consistently in both 2017 and 2018. All eligible hospitalizations were included, and no sampling was performed.

The unit of analysis was the hospitalization recorded in the hospital reporting system. The available aggregated data did not permit the identification of repeated hospitalizations involving the same patient; therefore, the terms “hospitalization” and “case” are used when reporting the study results.

Following identification of eligible major surgical hospitalizations, cases were categorized using the Diagnosis-Related Group (DRG) framework available in the institutional reporting database. Major Diagnostic Categories (MDCs) were used to describe the overall distribution of the surgical population. Adjacent Diagnosis-Related Groups (A-DRGs), which combine DRGs representing similar clinical conditions but different levels of complexity or resource consumption, were used for the more detailed analyses.

The A-DRG categories individually reported in the source analysis were retained for the category-level analyses. Categories that were not individually reported in the source dataset were combined as “Other A-DRGs”. The analyzed population included orthopedic, digestive, vascular, urinary tract, hepatobiliary, gynecological, neurological, infectious, and other major surgical categories.

Before analysis, internal arithmetic consistency checks were performed across annual totals and A-DRG subtotals. For category-level analyses, the residual “Other A-DRGs” category was calculated as the annual total minus the sum of the individually listed A-DRGs. This procedure ensured that each hospitalization contributed once to the A-DRG distribution.

### 2.3. Definition of Documented Anemia and Recorded Management Variables

Documented anemia was identified from primary or secondary diagnoses recorded in the hospital discharge records. The anemia status was checked against hemoglobin results documented in the medical records and recorded as present or absent. Individual hemoglobin values were not retained in the aggregated dataset; only the binary anemia status was available for analysis. Anemia diagnoses were identified using the International Classification of Diseases, Tenth Revision (ICD-10), as implemented in the institutional hospital discharge database. Both primary and secondary diagnoses were considered. Iron-deficiency anemia was identified using ICD-10 codes D50.0, D50.1, D50.8, and D50.9, whereas other anemia diagnoses were identified using codes D51–D64. Hospitalizations with at least one eligible anemia diagnosis recorded as either a primary or secondary diagnosis were counted once in the overall documented-anemia category. The hemoglobin-based check was not a formal validation study of ICD-10 coding accuracy, and diagnostic accuracy measures were not calculated. The ICD-10 classification of iron-deficiency anemia was not independently confirmed using biochemical measures of iron status, such as serum ferritin or transferrin saturation. The source dataset distinguished hospitalizations with an iron-deficiency anemia code from those with other documented anemia diagnoses. Accordingly, the category of anemia without documented iron deficiency should not be interpreted as confirmed absence of iron deficiency. In 2017, 1059 hospitalizations were associated with anemia without documented iron deficiency and 455 with anemia with documented iron deficiency. The corresponding numbers in 2018 were 1015 and 424, respectively. Because the source analysis identified substantial differences between hospital departments in the coding of anemia with versus without iron deficiency, the main analyses used total documented anemia, irrespective of whether iron deficiency was specifically coded. Total documented anemia therefore comprised 1514 hospitalizations in 2017 and 1439 in 2018. To our knowledge, the accuracy of these anemia codes has not been formally evaluated in this hospital or reported in a validation study from another Romanian hospital.

Although laboratory-based anemia criteria and selected biochemical measurements were reported in the previous publication using the same institutional surgical population [13]. For the present secondary analysis, individual hemoglobin values were not retained in the aggregated dataset, although hemoglobin results had been reviewed to document the anemia status as present or absent. Serum iron, ferritin, transferrin saturation, and the timing of anemia recognition relative to surgery were unavailable. Consequently, anemia severity could not be assessed, and coded iron-deficiency anemia could not be independently validated using biochemical criteria. Documented anemia should therefore not be interpreted as equivalent to systematically diagnosed preoperative anemia.

The recorded management variables were ferric carboxymaltose (FCM) administration and red blood cell (RBC) transfusion. FCM administration was identified from institutional medication-consumption records, whereas RBC transfusion was identified from the corresponding hospital transfusion records. Both variables were recorded at the hospitalization level and expressed as the number of hospitalizations with at least one documented administration of FCM or RBC transfusion, respectively. Hospitalizations with repeated FCM administrations or multiple RBC transfusions were counted only once for each corresponding variable. Thus, the reported counts do not represent the number of FCM doses, medication vials, individual administrations, or transfused RBC units. RBC transfusion was defined as the administration of at least one red blood cell component during hospitalization; transfusions involving plasma, platelets, or other blood components without RBC administration were not included. The aggregated dataset did not provide information on cumulative FCM doses, the number of RBC units administered, or the timing of either intervention relative to surgery. Both variables were evaluated across the entire major-surgery population, irrespective of documented anemia status.

Because the aggregated FCM and transfusion counts could not be consistently linked to documented anemia at the individual level, and because some hospitalizations could include both FCM administration and transfusion, these variables were not treated as mutually exclusive treatment groups. They were also not considered direct measures of PBM adherence, treatment appropriateness, or therapeutic effectiveness.

Individual classification according to documented anemia, FCM administration, and transfusion status was available only for the detailed analysis of the selected orthopedic A-DRGs I108 and I103. For these orthopedic subgroups, the source analysis provided separate management categories based on the presence or absence of documented anemia, FCM administration, and blood transfusion, including cases receiving both interventions. This more detailed classification was used for the exploratory orthopedic analyses. Equivalent hospitalization-level classification was not available for the remaining surgical A-DRGs. Accordingly, the total number of FCM-treated or transfused hospitalizations with documented anemia could not be established for the complete surgical cohort. Therefore, mutually exclusive management groups were constructed only for this exploratory orthopedic analysis.

### 2.4. Study Outcomes

The primary outcomes were the prevalence of documented anemia and the frequencies of recorded FCM administration and blood transfusion among major-surgery hospitalizations. These outcomes were described for 2017 and 2018 in the overall surgical population and across individual A-DRG categories.

Year-to-year comparisons were performed to assess whether the frequencies of documented anemia, recorded FCM administration, and recorded blood transfusion differed between 2017 and 2018. The distribution of these variables across A-DRG categories was examined descriptively to identify variation between major surgical settings.

A secondary exploratory analysis evaluated hospital resource utilization among hospitalizations with documented anemia in two orthopedic A-DRGs for which individual management-group data were available: I108, comprising other hip and femoral procedures, and I103, comprising hip replacement or revision of total hip arthroplasty.

Within each orthopedic A-DRG and study year, hospitalizations with documented anemia were classified into four mutually exclusive recorded management groups: no FCM/no transfusion, FCM/no transfusion, no FCM/transfusion, and FCM/transfusion. Hospital length of stay (LOS), intensive care unit (ICU) stay, and duration of surgery were summarized within these groups.

The exploratory orthopedic resource-utilization analysis was performed using a common analytical subset for hospital length of stay (LOS), intensive care unit (ICU) stay, and surgical duration. During the original data-preparation process, outlying observations were identified using the interquartile range (IQR) method (Tukey’s fences), with values below Q1 − 1.5 × IQR or above Q3 + 1.5 × IQR considered outliers. A total of 16 hospitalizations were excluded during data preparation, predominantly because of outlying resource-utilization values. These exclusions comprised 11 hospitalizations from A-DRG I108 in 2017, one from I108 in 2018, and four from I103 in 2017. No hospitalizations were excluded from I103 in 2018. The resulting common analytical subset comprised 577 of the 593 hospitalizations with documented anemia (97.3%). Filtering was performed at the hospitalization level, resulting in a common analytical subset of 577 hospitalizations for all three resource-utilization outcomes. Thus, the 16 excluded hospitalizations were omitted from each outcome analysis rather than representing separate sets of exclusions. The original data-preparation records did not specify how many hospitalizations were identified as outliers for each individual outcome.

### 2.5. Statistical Analysis

Categorical variables were summarized as frequencies and percentages. The prevalence of documented anemia was calculated using the total number of major-surgery hospitalizations in the corresponding year or A-DRG category as the denominator. Recorded FCM administration and blood transfusion were calculated using the same population-level denominators.

Comparisons between 2017 and 2018 were performed separately for documented anemia, recorded FCM administration, and recorded blood transfusion using Pearson’s chi-square test. Effect sizes were expressed as odds ratios (ORs) with 95% confidence intervals (CIs), with 2017 used as the reference year. An OR greater than 1 indicated higher odds of the corresponding outcome in 2018, whereas an OR below 1 indicated lower odds in 2018.

To account for differences in the distribution of surgical categories between 2017 and 2018, additional exploratory analyses were performed using the Cochran–Mantel–Haenszel method, stratified by the A-DRG category. Separate analyses were conducted for recorded FCM administration and blood transfusion. Common odds ratios and corresponding 95% confidence intervals were estimated across the 18 mutually exclusive A-DRG categories. The homogeneity of stratum-specific odds ratios was assessed using the Breslow–Day test. These analyses accounted for the surgical-category composition but did not adjust for individual clinical characteristics or differences within A-DRG categories.

The initial year-to-year comparisons were unadjusted, whereas the additional Cochran–Mantel–Haenszel analyses accounted for differences in the surgical-category composition. However, neither approach could account for differences in patient-level characteristics or surgical complexity within individual A-DRG categories. A-DRG-level findings were interpreted descriptively, and no formal between-category hypothesis testing was performed. Because repeated hospitalizations involving the same patient could not be identified, all hospitalizations were treated as independent observations in the statistical analyses. Consequently, potential within-patient correlation could not be accounted for, and the reported confidence intervals and *p*-values may be affected by violations of the independence assumption.

For the exploratory orthopedic analysis, hospital LOS, ICU stay, and surgical duration were summarized using means and standard deviations when these measures were available in the source report. No inferential comparisons were performed because several management groups contained very small numbers of observations and because complete measures of variability were not available for all groups. Given the limited subgroup sizes, incomplete reporting of standard deviations, and the exclusion of extreme observations during data preparation, the orthopedic resource-utilization analyses were considered strictly descriptive and hypothesis-generating. No conclusions regarding differences in treatment effectiveness or clinical outcomes were drawn from these comparisons.

All statistical tests were two-sided, and a *p* value < 0.05 was considered statistically significant. Statistical analyses were performed using IBM SPSS Statistics for Windows, version 29.0 (IBM Corp., Armonk, NY, USA).

### 2.6. Ethical Considerations

The study was conducted in accordance with the Declaration of Helsinki and approved by the Ethics Committee of “Victor Babeș” University of Medicine and Pharmacy, Timișoara, Romania (Approval No. 68, dated 1 October 2025). The ethical approval was obtained specifically for the retrospective secondary analysis of previously collected institutional data covering the period 2017–2018. No prospective patient recruitment or additional clinical interventions were performed as part of the present study.

## 3. Results

### 3.1. Study Population and Distribution of Major Surgical Procedures

The analysis included 10,571 major-surgery hospitalizations, comprising 5370 cases in 2017 and 5201 in 2018. The total annual number of hospitalizations decreased by 3.1% between the two years. However, the distribution across the MDCs changed, most notably for musculoskeletal and connective tissue disorders, which decreased from 1478 hospitalizations in 2017 to 935 in 2018. Renal and urinary tract, digestive, circulatory, and hepatobiliary categories were also consistently represented in both years (Table 1).

### 3.2. Prevalence and Distribution of Documented Anemia

Documented anemia was recorded in 1514 of 5370 major-surgery hospitalizations in 2017 (28.2%) and 1439 of 5201 in 2018 (27.7%). The overall prevalence was therefore similar between the two study years.

**Figure 1:**
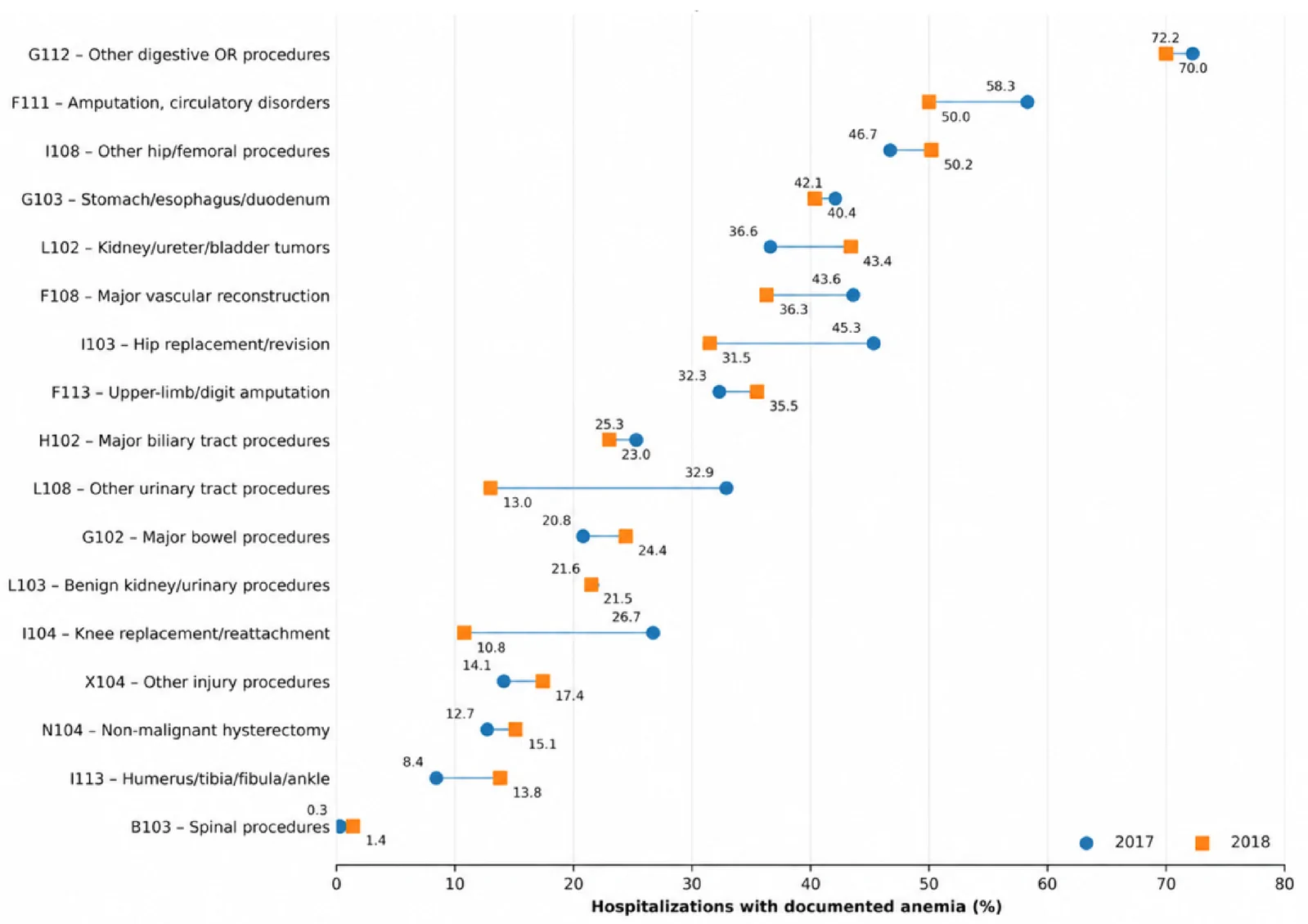
Prevalence of documented anemia across major surgical categories in 2017 and 2018. The figure shows the proportion of hospitalizations with a documented primary or secondary diagnosis of anemia within each adjacent Diagnosis-Related Group (A-DRG). Connected markers represent the difference in documented anemia prevalence between the two study years. Categories are ordered according to their overall prevalence of documented anemia to facilitate visual comparison. A-DRG, adjacent Diagnosis-Related Group.

Considerable variation was observed across A-DRG categories. The highest prevalence was recorded for G112, comprising other operating-room procedures involving the digestive system, in both 2017 and 2018 (72.2% and 70.0%, respectively). High prevalences were also observed in F111 amputations for circulatory disorders, I108 hip and femoral procedures, G103 stomach, esophageal and duodenal procedures, F108 vascular reconstruction, and I103 hip replacement or revision. Marked year-to-year differences were observed in selected categories, including I103, L108, and I104, despite the similar overall prevalence of documented anemia (Table 2 and Figure 1).

### 3.3. Year-to-Year Differences in Documented Anemia and Recorded Management Events

**Figure 2:**
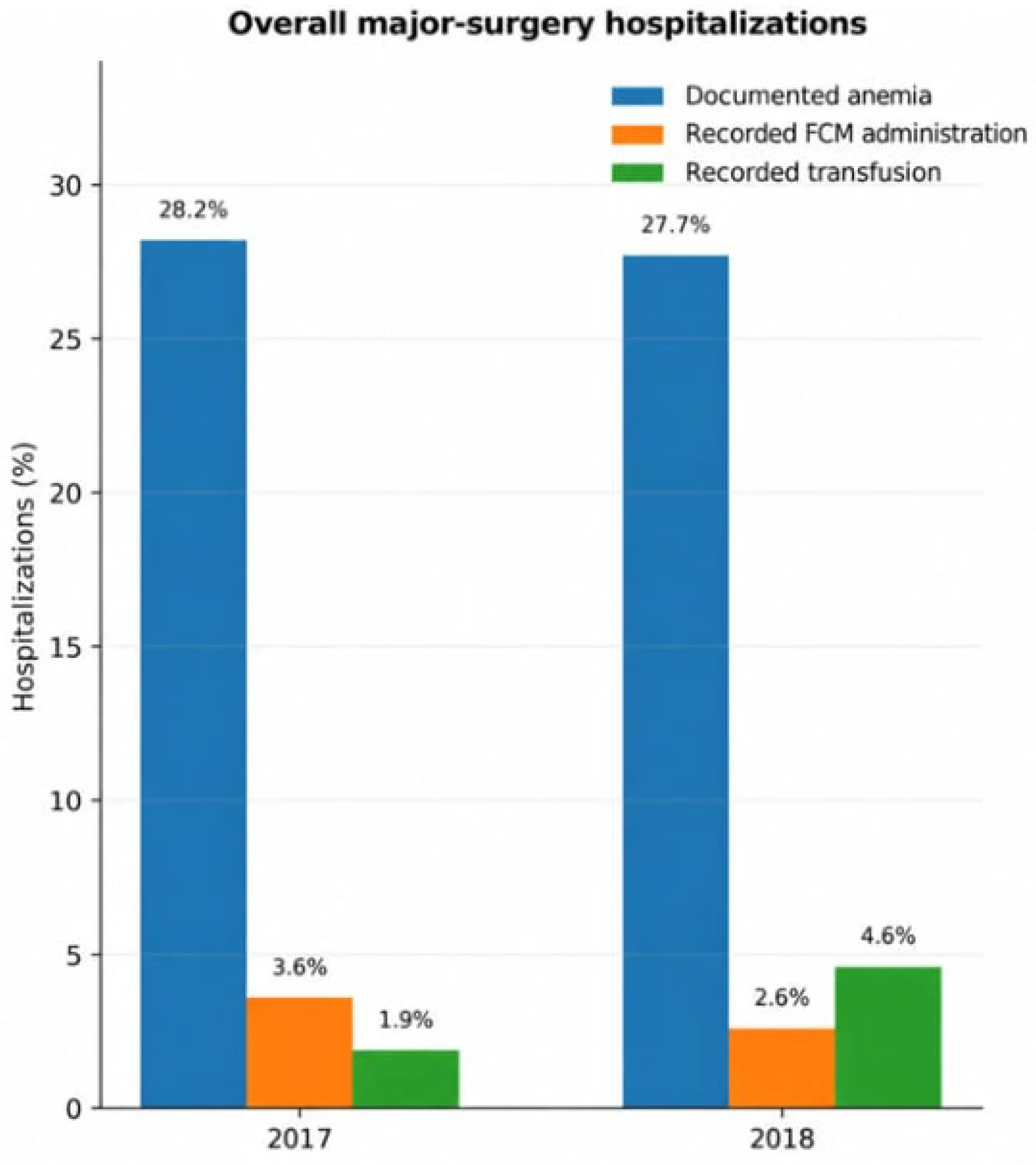
Documented anemia and recorded management events among major-surgery hospitalizations in 2017 and 2018. Percentages were calculated using all major-surgery hospitalizations in the corresponding year as the denominator. FCM administration and blood transfusion represent recorded events in the overall surgical population and were not restricted to hospitalizations with documented anemia. These variables should not be interpreted as mutually exclusive treatment categories. FCM, ferric carboxymaltose.

The prevalence of documented anemia did not differ significantly between 2017 and 2018 (28.2% vs. 27.7%; OR 0.97, 95% CI 0.89–1.06; *p* = 0.547). In contrast, recorded FCM administration decreased from 3.6% to 2.6% (absolute difference, −1.0 percentage point; OR 0.70, 95% CI 0.56–0.88; *p* = 0.002), whereas recorded blood transfusion increased from 1.9% to 4.6% (absolute difference, 2.7 percentage points; OR 2.46, 95% CI 1.95–3.11; *p* < 0.001). These unadjusted findings describe different year-to-year patterns in documented anemia and recorded management events but do not establish that the reduction in FCM administration was related to the increase in transfusion (Table 3 and Figure 2). Importantly, the reported FCM and transfusion frequencies were calculated using all major-surgery hospitalizations as denominators, irrespective of documented anemia status. Because the aggregated data did not permit consistent hospitalization-level linkage between anemia diagnoses and recorded interventions, these findings should not be interpreted as mutually exclusive treatment pathways or as evidence that patients with documented anemia received either intervention. Additional analyses stratified by the surgical A-DRG category attenuated the association between study year and recorded FCM administration. The unadjusted odds ratio of 0.70 (95% CI 0.56–0.88) increased to a pooled Cochran–Mantel–Haenszel odds ratio of 0.86 (95% CI 0.68–1.08; *p* = 0.190), indicating that the association was no longer statistically significant after accounting for the surgical-category composition. In contrast, the association between study year and recorded blood transfusion remained statistically significant after stratification (pooled OR 2.49, 95% CI 1.97–3.14; *p* < 0.001), compared with an unadjusted OR of 2.46. Tests of homogeneity showed no statistically significant heterogeneity for FCM administration (*p* = 0.196), whereas significant heterogeneity was observed for blood transfusion (*p* < 0.001). Therefore, the pooled transfusion estimate should be interpreted cautiously, as year-to-year associations varied across surgical categories (Table 4).

### 3.4. Distribution of Documented Anemia and Recorded Management Events Across Surgical Categories

The distribution of documented anemia, FCM administration, and blood transfusion varied considerably across A-DRG categories. Categories with a high prevalence of documented anemia did not consistently have high population-level frequencies of recorded FCM administration or transfusion.

In I103 hip replacement or revision procedures, documented anemia decreased from 45.3% in 2017 to 31.5% in 2018, recorded FCM administration decreased from 12.1% to 7.9%, and transfusion increased from 0.7% to 16.1%. In I108 hip and femoral procedures, documented anemia increased slightly from 46.7% to 50.2%, recorded FCM administration decreased from 12.1% to 10.7%, and transfusion increased from 2.2% to 12.9%. Other categories, including G112 and F111, had a high prevalence of documented anemia but comparatively low population-level frequencies of recorded FCM administration and transfusion.

**Figure 3:**
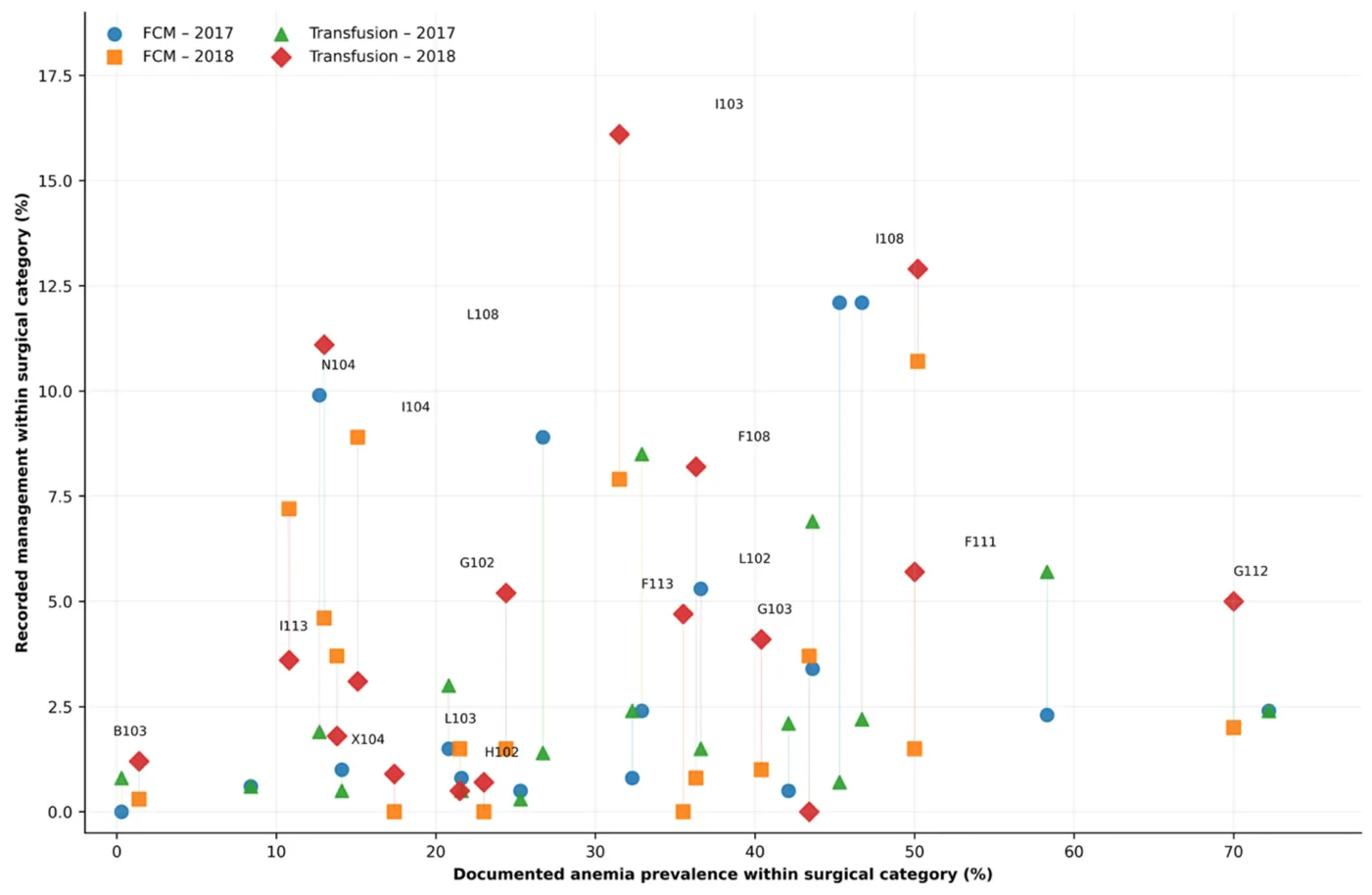
Distribution of documented anemia and recorded management events across A-DRG categories in 2017 and 2018. Each point represents the percentage of hospitalizations with the corresponding recorded event within an A-DRG category and study year. The figure provides a descriptive comparison and does not represent individual-level associations between documented anemia, FCM administration, and blood transfusion. All percentages were calculated using the total number of hospitalizations within the corresponding A-DRG and year as the denominator. A-DRG, adjacent Diagnosis-Related Group; FCM, ferric carboxymaltose.

These findings are descriptive and should be interpreted in the context of differences in surgical case mix and the absence of complete individual linkage between documented anemia and the recorded management events (Table 5 and Figure 3).

### 3.5. Clinical Resource Utilization According to Anemia-Management Pattern

Case-level resource-utilization data were available for hospitalizations with documented anemia in two orthopedic A-DRGs: I108 and I103. Mean hospital LOS, ICU stay, and surgical duration varied across the recorded management groups, but no consistent pattern was observed across A-DRGs or study years. Several groups contained fewer than five observations, particularly those involving both FCM administration and transfusion. In particular, several management groups comprised only two or three hospitalizations, making their mean values highly sensitive to individual observations. Consequently, small differences in hospital length of stay, ICU stay, or surgical duration should not be interpreted as reliable differences between management groups. No inferential comparisons were therefore performed, and the results presented in Table 6 were interpreted as exploratory descriptive findings. Of the 593 hospitalizations with documented anemia in the two orthopedic A-DRGs, 577 (97.3%) were retained in the common analytical subset used for hospital LOS, ICU stay, and surgical duration. The remaining 16 hospitalizations (2.7%) were excluded during the original data-preparation process, predominantly because of outlying observations identified using the IQR method. Specifically, 11 hospitalizations were excluded from I108 in 2017, one from I108 in 2018, and four from I103 in 2017, whereas no exclusions occurred in I103 in 2018. The original data-preparation records did not specify how many of the 16 excluded hospitalizations were independently flagged as outliers for each individual outcome. These exclusions explain the differences between the anemia totals reported in Table 2 and the analytical subgroup totals presented in Table 6.

Within the available analytical subset of orthopedic A-DRGs I108 and I103, 79 hospitalizations with documented anemia were classified as having received FCM, including eight hospitalizations that also received blood transfusion. Similarly, 47 hospitalizations with documented anemia had recorded transfusion, including the same eight cases receiving both interventions. Overall, 118 distinct hospitalizations with documented anemia were classified as receiving at least one of these interventions. These counts refer to the available orthopedic analytical subset after the exclusions applied in the source analysis and should not be interpreted as complete treatment totals for the overall surgical population.

## 4. Discussion

This retrospective study described documented anemia, recorded FCM administration, and recorded blood transfusion across 10,571 major-surgery hospitalizations. Documented anemia was recorded in approximately 28% of hospitalizations and did not differ significantly between 2017 and 2018. In contrast, recorded FCM administration decreased, whereas blood transfusion increased. Considerable variation was also observed across surgical categories, particularly in selected orthopedic A-DRGs. Because the population-level management records could not be consistently linked to documented anemia at the individual level, these findings describe administrative and management patterns rather than individual treatment pathways.

The present findings extend the previously published analysis of this institutional surgical population by shifting the focus from laboratory-defined anemia and individual treatment-related outcomes to the distribution of documented anemia and recorded management events across surgical categories. The additional A-DRG-stratified analyses provide information on the extent to which differences in surgical case mix may contribute to year-to-year variation in recorded FCM administration and transfusion. These findings should be regarded as complementary to, rather than independent confirmation of, the earlier study because both analyses originate from the same institutional surgical population. Importantly, although anemia diagnoses were checked against hemoglobin results, individual hemoglobin values were not retained in the dataset; therefore, the present analysis cannot report hemoglobin distributions or anemia severity. The clinical relevance of this secondary analysis lies in its ability to identify procedure-specific variation that may be obscured by overall institutional estimates. In particular, the category-stratified analyses provide a more informative interpretation of year-to-year changes by accounting for differences in surgical case mix, while the orthopedic subgroup analyses highlight areas in which more detailed evaluation of anemia recognition and management may be warranted. These findings may inform the prioritization of targeted PBM audits and quality-improvement strategies, although they cannot establish treatment appropriateness, effectiveness, or causal relationships.

Documented anemia was recorded in 28.2% of major-surgery hospitalizations in 2017 and 27.7% in 2018. Comparisons with studies reporting individual hemoglobin measurements should be made cautiously because the present analysis retained anemia status as a binary variable and did not retain the individual hemoglobin values or the timing of their measurement. This is clinically relevant because perioperative anemia is not simply a laboratory abnormality. It reduces arterial oxygen content and, when compensatory increases in cardiac output and oxygen extraction become insufficient, may compromise tissue oxygen delivery. The problem becomes particularly relevant during major surgery, where blood loss, hemodilution, inflammation, and postoperative suppression of erythropoiesis may further aggravate a pre-existing deficit [14]. Accordingly, current international recommendations consider preoperative anemia a modifiable risk factor and advocate systematic screening before most major procedures, followed by investigation of its underlying cause rather than correction of hemoglobin alone [15].

Yet our results also show why a single prevalence estimate tells only part of the story. Documented anemia was unevenly distributed across surgical categories. Rates approached 70% in some major digestive procedures and were also high in vascular and several orthopedic groups, whereas other categories showed much lower values. This heterogeneity is biologically plausible. Patients undergoing gastrointestinal surgery may have chronic occult blood loss, malignancy, impaired nutritional intake, or inflammation-mediated iron restriction [16,17]. Vascular patients frequently combine chronic inflammation, renal impairment and substantial comorbidity. Orthopedic populations, particularly those involving the hip and femur, include both elective degenerative disease and acute presentations in which blood loss and limited time for preoperative optimization may coexist [16,18]. In inflammatory states, increased hepcidin activity reduces intestinal iron absorption and sequesters iron within macrophages, producing iron-restricted erythropoiesis even when total body iron stores are not depleted [19]. Thus, apparently similar patterns of documented anemia across surgical groups may reflect different underlying mechanisms. This is precisely why contemporary PBM recommendations emphasize etiological assessment, including evaluation of iron status, rather than treating all perioperative anemia as a uniform entity [16,18]. However, differences in diagnostic coding, case mix, surgical urgency, and the timing of anemia recognition may also have contributed to the observed variation.

A notable finding was the change in recorded management events despite the similar overall prevalence of documented anemia. Recorded FCM administration decreased from 3.6% to 2.6%, whereas recorded blood transfusion increased from 1.9% to 4.6%. Importantly, the additional category-stratified analyses suggested that differences in surgical case mix contributed to the observed reduction in recorded FCM administration, as the association was attenuated and no longer statistically significant after stratification by A-DRG. Conversely, the association between study year and recorded transfusion remained statistically significant after stratification. However, substantial heterogeneity across surgical categories indicates that this pooled association should not be interpreted as a uniform change across all procedures. Moreover, residual confounding by patient characteristics, surgical urgency, anemia severity, and institutional practices cannot be excluded. However, these changes cannot be interpreted as a shift from FCM treatment to transfusion because the aggregated variables were not restricted to the same patients, were not mutually exclusive, and were not adjusted for differences in surgical case mix. Nevertheless, this divergence is noteworthy from a PBM perspective. Contemporary PBM strategies aim to move anemia management upstream by identifying anemia and iron deficiency early, treating reversible causes when feasible, minimizing perioperative blood loss, and reserving transfusion for situations in which it is clinically indicated [20,21]. International consensus recommendations similarly support the early identification, etiological investigation, and treatment of anemia before major elective surgery [15,21]. The present findings demonstrate year-to-year differences in recorded management events; however, the available data cannot determine whether these differences resulted from changes in clinical practice, surgical case mix, diagnostic coding, treatment availability, transfusion thresholds, or patient severity.

The role of intravenous iron within this pathway is more nuanced than that of a simple alternative to transfusion. FCM can replenish iron stores rapidly and may be particularly useful when the time available before surgery is limited. Nevertheless, its effectiveness depends on the presence of iron deficiency, the inflammatory status, treatment timing, ongoing blood loss, and the interval available for erythropoiesis, while the evidence regarding major perioperative outcomes remains inconsistent [22]. In the PREVENTT randomized controlled trial, preoperative intravenous iron increased hemoglobin concentrations before major abdominal surgery but did not significantly reduce the risk of blood transfusion or death, or the number of transfusions, compared with the placebo [17]. This finding is important when interpreting our results. A lower frequency of recorded FCM administration cannot necessarily be equated with poorer care, just as greater FCM use cannot automatically be assumed to prevent transfusion. The absence of ferritin, transferrin saturation, inflammatory markers, and individual hemoglobin trajectories from our dataset prevented the assessment of treatment eligibility and response.

At the same time, the broader PBM literature suggests that the greatest benefit may arise from coordinated, multimodal programs rather than from any single intervention. A systematic review and meta-analysis of 17 studies involving 235,779 surgical patients found that multimodal PBM implementation was associated with lower transfusion rates, shorter hospital stays, fewer postoperative complications, and lower mortality [23]. Hospital-level implementation studies have similarly demonstrated improvements in transfusion appropriateness following the introduction of multidisciplinary PBM programs and clinician feedback [24]. Against this background, the marked variation between surgical categories in our study becomes particularly relevant. A-DRG categories with similarly high prevalences of documented anemia showed different frequencies of recorded FCM administration and blood transfusion. These differences may reflect procedure type, surgical urgency, case mix, specialty-specific practices, institutional factors, or variation in documentation.

The orthopedic findings illustrate this issue particularly well. In hip replacement or revision procedures, recorded FCM administration decreased between the two years, whereas blood transfusion increased markedly. A similar, although less pronounced, pattern was observed in other hip and femoral procedures. These changes were not accompanied by a comparable change in the overall prevalence of documented anemia, although the available data cannot establish their underlying causes. They may reflect differences in case mix, perioperative blood loss, surgical urgency, transfusion thresholds, or the time available for preoperative anemia correction. Published evidence on intravenous iron in orthopedic surgery remains heterogeneous. A recent meta-analysis reported lower transfusion rates following high-dose intravenous iron administration in patients undergoing total hip or knee arthroplasty [25]. In contrast, a meta-analysis restricted to randomized trials in patients undergoing surgery for femoral fractures found no statistically significant reduction in transfusion exposure or other major clinical outcomes [26]. These contrasting findings indicate that treatment effects may depend on the orthopedic population, surgical setting, timing of administration, and study design. Our descriptive findings therefore support further procedure-specific evaluation of PBM pathways, particularly in orthopedic categories showing substantial year-to-year variation in recorded management events.

The aggregated nature of the data also prevented reliable estimation of the proportion of hospitalizations with documented anemia in which neither FCM administration nor blood transfusion was recorded. Population-level FCM and transfusion counts included events recorded outside the coded-anemia group, and some hospitalizations may have included both interventions. Therefore, subtracting these totals from the number of hospitalizations with documented anemia would not produce a valid, mutually exclusive untreated group. This limitation emphasizes the need for patient-level linkage when evaluating the clinical pathway following anemia recognition.

Finally, the exploratory orthopedic analysis showed no consistent relationship between recorded management patterns and hospital or ICU length of stay. This finding is not unexpected because length of stay is a multifactorial outcome influenced by patient comorbidity, functional status, surgical procedure, perioperative complications, organizational factors, and the timing of surgery [27]. Treatment selection may also be influenced by clinical severity, creating a substantial risk of confounding by indication. Similar uncertainty has been reported in the broader literature: although PBM interventions can reduce exposure to red blood cell transfusion, randomized evidence has not consistently demonstrated corresponding benefits in mortality or other major clinical outcomes [28]. Our data should therefore not be used to infer a causal effect of FCM administration or blood transfusion on hospital resource utilization. Rather, they underline the difficulty of evaluating individual PBM components outside a structured clinical pathway. Taken together, the findings support a transition from simply documenting anemia toward systematically investigating its underlying cause, identifying patients most likely to benefit from intervention, and integrating these decisions into procedure-specific PBM pathways.

### 4.1. Strengths and Limitations

The main strength of this study is the inclusion of all 10,571 major-surgery hospitalizations available in the institutional dataset across two consecutive years. The population covered a broad range of surgical categories, allowing the distribution of documented anemia, FCM administration, and transfusion to be described across different clinical settings.

Several limitations must be considered. First, anemia diagnoses recorded in the discharge data were checked against hemoglobin results documented in the medical records, and anemia status was recorded as present or absent. Individual hemoglobin values were not retained in the aggregated dataset, so anemia severity could not be assessed. This hemoglobin-based check was not a formal validation study of ICD-10 coding accuracy, and diagnostic accuracy measures were not calculated. To our knowledge, the accuracy of these codes has not been formally evaluated in this hospital or reported in a validation study from another Romanian hospital. Iron-deficiency anemia could not be independently confirmed using biochemical measures of iron status.

Second, differences in anemia coding were observed between hospital departments. Total documented anemia was used to reduce the influence of variation in iron-deficiency-specific coding, but variation in the overall recognition and documentation of anemia could not be excluded.

Third, population-level FCM and transfusion records could not be consistently linked to documented anemia at the individual level. Some management events occurred among hospitalizations without a documented anemia diagnosis, and some hospitalizations included both FCM administration and transfusion. Consequently, these variables cannot be interpreted as mutually exclusive treatment groups or used to determine the proportion of all hospitalizations with documented anemia that received neither intervention.

Fourth, although additional analyses stratified by the surgical A-DRG category were performed, these analyses could not account for patient-level confounding or differences in clinical characteristics within surgical categories. The distribution of surgical categories changed between 2017 and 2018, including a substantial reduction in musculoskeletal hospitalizations, potentially contributing to the observed differences in FCM administration and transfusion. Information regarding surgical urgency, comorbidities, anemia severity, perioperative blood loss, transfusion thresholds, number of units administered, postoperative complications, readmission, and mortality was unavailable. Furthermore, the aggregated records did not provide information on cumulative FCM doses, repeated administrations, or the timing of FCM administration and transfusion relative to surgery. Consequently, treatment intensity and perioperative timing could not be evaluated. Furthermore, significant heterogeneity in the category-specific transfusion associations indicates that the pooled stratified estimate may not adequately represent the associations observed across individual surgical categories. Additionally, repeated hospitalizations involving the same patient could not be identified in the available dataset, potentially violating the assumption of statistical independence and affecting standard error estimates, confidence intervals, and *p*-values. Consequently, the findings should be interpreted as exploratory associations rather than evidence of changes in clinical practice or treatment effectiveness.

Finally, individual management-group and resource-utilization data were available only for selected orthopedic A-DRGs. The resource-utilization analysis was based on a common analytical subset of 577 hospitalizations after the exclusion of 16 observations during data preparation, predominantly because of outlying values identified using the IQR method. The same 16 hospitalizations were excluded from all three resource-utilization analyses; however, the original data-preparation records did not specify how many were independently flagged as outliers for hospital LOS, ICU stay, or surgical duration. Consequently, the relative contribution of extreme values in hospital LOS, ICU stay, and surgical duration to the exclusion process remains uncertain. Exclusion of extreme observations may have influenced the reported averages and introduced selection bias. Furthermore, several management groups included only two or three hospitalizations, making the reported means highly sensitive to individual observations. These findings should therefore be considered exploratory and cannot support causal conclusions regarding the effects of FCM administration or transfusion on hospital resource utilization.

Although major surgery was defined using clinical and procedural criteria, eligibility was not based on a predefined list of procedure codes. Therefore, some variability in the interpretation of surgical complexity and anticipated blood loss cannot be excluded. This should be considered when assessing the reproducibility of the selection process. An additional limitation is the historical nature of the dataset, which covers 2017–2018. Since that period, PBM recommendations, perioperative anemia-management strategies, and transfusion practices may have evolved. Furthermore, changes in institutional protocols, surgical case mix, clinical staffing, treatment availability, or documentation practices during or after the study period could have influenced the observed patterns. However, systematic information regarding such institutional changes was not available in the dataset used for this secondary analysis. Consequently, the findings should be interpreted as a historical institutional assessment rather than a representation of current clinical practice, and their generalizability to contemporary PBM implementation is limited.

### 4.2. Clinical Implications and Future Directions

The findings show that documented anemia and recorded management events were unevenly distributed across major surgical categories. However, administrative indicators considered separately cannot provide a complete assessment of PBM implementation. A valid evaluation requires patient-level linkage between anemia recognition, laboratory characterization, treatment selection, treatment timing, transfusion exposure, and clinical outcomes.

The marked variation observed in selected orthopedic A-DRGs identifies these categories as potential priorities for prospective clinical audit. Such an audit should include preoperative hemoglobin, ferritin, transferrin saturation, inflammatory markers, surgical urgency, estimated blood loss, transfusion indications, treatment timing, and postoperative outcomes.

Future studies should use prospectively defined patient-level datasets and adjust comparisons for case mix, anemia severity, comorbidities, and procedural complexity. This would permit evaluation of whether differences in recorded management reflect differences in clinical need, institutional practice, or PBM implementation.

## 5. Conclusions

Documented anemia was common among major-surgery hospitalizations, affecting approximately 28% of cases in both 2017 and 2018. Recorded FCM administration and red blood cell transfusion varied across surgical categories and between study years. After stratification by surgical A-DRG category, the year-to-year association with FCM administration was attenuated, whereas the association with transfusion persisted, although substantial between-category heterogeneity was observed. These historical findings highlight variation in recorded anemia-management indicators but do not establish treatment appropriateness or effectiveness. Future patient-level studies linking laboratory-defined anemia, treatment decisions, and clinical outcomes are needed to evaluate Patient Blood Management implementation.

## Figures and Tables

**Table 1:** Distribution of major surgical procedures according to surgical/diagnostic category, 2017–2018.

MDC	Major Diagnostic Category	2017, *n* (%)	2018, *n* (%)	Total, *n* (%)
8	Musculoskeletal system and connective tissue disorders	1478 (27.5)	935 (18.0)	2413 (22.8)
11	Kidney and urinary tract disorders	832 (15.5)	852 (16.4)	1684 (15.9)
6	Digestive system disorders	790 (14.7)	893 (17.2)	1683 (15.9)
5	Circulatory system disorders	666 (12.4)	748 (14.4)	1414 (13.4)
7	Hepatobiliary system and pancreatic disorders	452 (8.4)	497 (9.6)	949 (9.0)
13	Female reproductive system disorders	369 (6.9)	405 (7.8)	774 (7.3)
1	Nervous system disorders	365 (6.8)	346 (6.7)	711 (6.7)
21	Injuries, poisoning and toxic effects of drugs	292 (5.4)	331 (6.4)	623 (5.9)
18	Infectious and parasitic diseases	63 (1.2)	83 (1.6)	146 (1.4)
12	Male reproductive system disorders	28 (0.5)	63 (1.2)	91 (0.9)
17	Neoplastic disorders (hematologic and solid tumors)	18 (0.3)	35 (0.7)	53 (0.5)
16	Blood, blood-forming organ and immunological disorders	17 (0.3)	13 (0.2)	30 (0.3)
Total		5370 (100)	5201 (100)	10,571 (100)

Percentages were calculated relative to the total number of major surgical cases recorded in each study year (*n* = 5370 in 2017 and *n* = 5201 in 2018). Overall percentages were calculated using the complete study population (*N* = 10,571). Abbreviations: MDC, Major Diagnostic Category.

**Table 2:** Prevalence of documented anemia according to major surgical category, 2017–2018.

A-DRG	Major Surgical Category	2017 Total, *n*	2017 Anemia, n (%)	2018 Total, *n*	2018 Anemia, *n* (%)
G112	Other operating-room procedures involving the digestive system	169	122 (72.2)	200	140 (70.0)
F111	Amputation for circulatory disorders, excluding upper limb and toe procedures	175	102 (58.3)	194	97 (50.0)
I108	Other hip and femoral procedures	448	209 (46.7)	233	117 (50.2)
G103	Procedures involving the stomach, esophagus, and duodenum	190	80 (42.1)	193	78 (40.4)
F108	Major vascular reconstruction procedures without cardiopulmonary bypass	291	127 (43.6)	366	133 (36.3)
L102	Major kidney, ureter, and bladder procedures for tumors	131	48 (36.6)	136	59 (43.4)
F113	Upper-limb and digit amputation for circulatory disorders	127	41 (32.3)	107	38 (35.5)
I103	Hip replacement or revision of total hip arthroplasty	404	183 (45.3)	267	84 (31.5)
L108	Other procedures for kidney and urinary tract disorders	82	27 (32.9)	108	14 (13.0)
I104	Knee replacement and reattachment procedures	146	39 (26.7)	83	9 (10.8)
H102	Major biliary tract procedures with or without malignancy	392	99 (25.3)	413	95 (23.0)
G102	Major small- and large-bowel procedures	337	70 (20.8)	402	98 (24.4)
L103	Major kidney, ureter, and bladder procedures for benign tumors	619	134 (21.6)	608	131 (21.5)
X104	Other procedures for injuries	199	28 (14.1)	235	41 (17.4)
N104	Hysterectomy for non-malignant conditions	213	27 (12.7)	225	34 (15.1)
I113	Humerus, tibia, fibula, and ankle procedures	178	15 (8.4)	109	15 (13.8)
B103	Spinal procedures	365	1 (0.3)	346	5 (1.4)
Other A-DRGs	Other major surgical procedures	904	162 (17.9)	976	251 (25.7)
Total	All major surgical procedures	5370	1514 (28.2)	5201	1439 (27.7)

Documented anemia prevalence was calculated as the proportion of hospitalizations with a primary or secondary diagnosis of anemia among all hospitalizations within the corresponding A-DRG category. Because substantial between-department variation was observed in the coding of anemia with versus without iron deficiency, total documented anemia was used for this analysis. Abbreviations: A-DRG, adjacent Diagnosis-Related Group.

**Table 3:** Documented anemia and recorded management events among major-surgery hospitalizations.

Variable	2017, *n*/*N* (%)	2018, *n*/*N* (%)	OR (95% CI), 2018 vs. 2017	*p*-Value
Documented anemia	1514/5370 (28.2)	1439/5201 (27.7)	0.97 (0.89–1.06)	0.547
Recorded FCM administration	195/5370 (3.6)	134/5201 (2.6)	0.70 (0.56–0.88)	0.002
Recorded blood transfusion	103/5370 (1.9)	239/5201 (4.6)	2.46 (1.95–3.11)	<0.001

ORs compare the odds of each outcome in 2018 with those in 2017. Percentages were calculated using all major-surgery hospitalizations in the corresponding year as the denominator. FCM administration and blood transfusion represent recorded events in the overall surgical population and were not restricted to hospitalizations with documented anemia. The variables should not be interpreted as mutually exclusive treatment groups. Abbreviations: CI, confidence interval; FCM, ferric carboxymaltose; OR, odds ratio.

**Table 4:** Unadjusted and surgical-category-stratified associations between study year and recorded management events.

Outcome	Unadjusted OR (95% CI)	A-DRG-Stratified OR (95% CI)	CMH *p*-Value	Homogeneity *p*-Value
Recorded FCM administration	0.70 (0.56–0.88)	0.86 (0.68–1.08)	0.190	0.196
Recorded blood transfusion	2.46 (1.95–3.11)	2.49 (1.97–3.14)	<0.001	<0.001

Odds ratios compare 2018 with 2017, using 2017 as the reference year. Stratified estimates were obtained using the Cochran–Mantel–Haenszel method across 18 mutually exclusive A-DRG categories. Homogeneity of stratum-specific odds ratios was assessed using the Breslow–Day test. The pooled transfusion estimate should be interpreted cautiously because of significant between-stratum heterogeneity. A-DRG, adjacent Diagnosis-Related Group; CI, confidence interval; CMH, Cochran–Mantel–Haenszel; FCM, ferric carboxymaltose; OR, odds ratio.

**Table 5:** Documented anemia and recorded management events according to major surgical category.

A-DRG	Major Surgical Category	2017 Anemia, *n* (%)	2017 FCM Administration, *n* (%)	2017 Transfusion, *n* (%)	2018 Anemia, *n* (%)	2018 FCM Administration, *n* (%)	2018 Transfusion, *n* (%)
L103	Major kidney, ureter, and bladder procedures for benign tumors	134 (21.6)	5 (0.8)	3 (0.5)	131 (21.5)	9 (1.5)	3 (0.5)
H102	Major biliary tract procedures with/without malignancy	99 (25.3)	2 (0.5)	1 (0.3)	95 (23.0)	0 (0.0)	3 (0.7)
G102	Major small- and large-bowel procedures	70 (20.8)	5 (1.5)	10 (3.0)	98 (24.4)	6 (1.5)	21 (5.2)
F108	Major vascular reconstruction without cardiopulmonary bypass	127 (43.6)	10 (3.4)	20 (6.9)	133 (36.3)	3 (0.8)	30 (8.2)
B103	Spinal procedures	1 (0.3)	0 (0.0)	3 (0.8)	5 (1.4)	1 (0.3)	4 (1.2)
I103	Hip replacement or revision of total hip arthroplasty	183 (45.3)	49 (12.1)	3 (0.7)	84 (31.5)	21 (7.9)	43 (16.1)
X104	Other procedures for injuries	28 (14.1)	2 (1.0)	1 (0.5)	41 (17.4)	0 (0.0)	2 (0.9)
I108	Other hip and femoral procedures	209 (46.7)	54 (12.1)	10 (2.2)	117 (50.2)	25 (10.7)	30 (12.9)
N104	Hysterectomy for non-malignant conditions	27 (12.7)	21 (9.9)	4 (1.9)	34 (15.1)	20 (8.9)	7 (3.1)
G112	Other operating-room procedures involving the digestive system	122 (72.2)	4 (2.4)	4 (2.4)	140 (70.0)	4 (2.0)	10 (5.0)
F111	Amputation for circulatory disorders, excluding upper limb and toe procedures	102 (58.3)	4 (2.3)	10 (5.7)	97 (50.0)	3 (1.5)	11 (5.7)
G103	Procedures involving the stomach, esophagus, and duodenum	80 (42.1)	1 (0.5)	4 (2.1)	78 (40.4)	2 (1.0)	8 (4.1)
L102	Major kidney, ureter, and bladder procedures for tumors	48 (36.6)	7 (5.3)	2 (1.5)	59 (43.4)	5 (3.7)	0 (0.0)
I113	Humerus, tibia, fibula, and ankle procedures	15 (8.4)	1 (0.6)	1 (0.6)	15 (13.8)	4 (3.7)	2 (1.8)
L108	Other kidney and urinary tract procedures	27 (32.9)	2 (2.4)	7 (8.5)	14 (13.0)	5 (4.6)	12 (11.1)
F113	Upper-limb and digit amputation for circulatory disorders	41 (32.3)	1 (0.8)	3 (2.4)	38 (35.5)	0 (0.0)	5 (4.7)
I104	Knee replacement and reattachment procedures	39 (26.7)	13 (8.9)	2 (1.4)	9 (10.8)	6 (7.2)	3 (3.6)
Other A-DRGs	Other major surgical procedures	162 (17.9)	14 (1.5)	15 (1.7)	251 (25.7)	20 (2.0)	45 (4.6)
Overall	All major surgical procedures	1514 (28.2)	195 (3.6)	103 (1.9)	1439 (27.7)	134 (2.6)	239 (4.6)

Percentages for documented anemia, FCM administration, and blood transfusion were calculated relative to the total number of hospitalizations within each corresponding A-DRG category and study year. Anemia represents any documented primary or secondary diagnosis of anemia, irrespective of whether iron deficiency was specifically coded. FCM administration and blood transfusion are presented as recorded management events and should not be interpreted as mutually exclusive categories. Abbreviations: A-DRG, adjacent Diagnosis-Related Group; FCM, ferric carboxymaltose.

**Table 6:** Length of stay, ICU stay and surgical duration according to anemia-management pattern in selected orthopedic procedures.

A-DRG	Year	Anemia-Management Pattern	*n*	Hospital LOS, Days, Mean (SD)	ICU Stay, Days, Mean (SD)	Surgical Duration, h, Mean
I108	2017	No FCM/no transfusion	166	16.46 (6.15)	1.39 (1.10)	1.56
	2017	FCM/no transfusion	27	17.26 (5.60)	2.50 (1.84)	1.44
	2017	No FCM/transfusion	2	14.00 (NR)	0.80 (NR)	1.00
	2017	FCM/transfusion	3	19.67 (NR)	2.60 (NR)	1.50
	2018	No FCM/no transfusion	83	14.80 (5.25)	1.16 (0.92)	1.87
	2018	FCM/no transfusion	14	15.43 (5.02)	2.02 (1.71)	1.50
	2018	No FCM/transfusion	17	13.00 (4.50)	1.22 (0.94)	2.10
	2018	FCM/transfusion	2	11.50 (NR)	1.50 (NR)	1.71
I103	2017	No FCM/no transfusion	155	17.14 (8.56)	1.72 (0.81)	1.92
	2017	FCM/no transfusion	22	16.41 (7.04)	1.67 (0.94)	2.08
	2017	No FCM/transfusion	2	11.50 (NR)	1.05 (NR)	2.83
	2017	FCM/transfusion	0	—	—	—
	2018	No FCM/no transfusion	55	16.93 (5.00)	1.25 (0.60)	2.03
	2018	FCM/no transfusion	8	18.00 (NR)	1.64 (NR)	2.20
	2018	No FCM/transfusion	18	17.33 (4.98)	1.24 (0.54)	2.47
	2018	FCM/transfusion	3	22.67 (NR)	1.33 (NR)	2.17

Analyses were restricted to hospitalizations with documented anemia in A-DRG I108 and I103. Within each A-DRG and study year, the management categories were mutually exclusive. A common analytical subset of 577 hospitalizations was used for all three resource-utilization outcomes. During data preparation, 16 hospitalizations were excluded, predominantly because of outlying values identified using the IQR method (Tukey’s fences). The exclusions comprised 11 hospitalizations for I108 in 2017, one for I108 in 2018, and four for I103 in 2017. The same 16 hospitalizations were excluded from the analyses of hospital LOS, ICU stay, and surgical duration. Thus, each outcome was summarized using the same 577 retained hospitalizations. The original data-preparation records did not specify the number of excluded hospitalizations flagged as outliers separately for hospital LOS, ICU stay, and surgical duration. Standard deviations are presented only when available in the source report. No inferential comparisons were performed because several groups contained very small numbers of observations. Mean values derived from groups comprising fewer than five hospitalizations should be interpreted with particular caution because they are highly sensitive to individual observations. These estimates are presented solely for exploratory descriptive purposes. Abbreviations: A-DRG, adjacent Diagnosis-Related Group; FCM, ferric carboxymaltose; ICU, intensive care unit; LOS, length of stay; NR, not reported.

## Data Availability

The data presented in this study are available upon request from the corresponding author. The data are not publicly available due to hospital policy.
